# High prevalence of intestinal protozoan infections among asymptomatic individuals in rural and urban communities in conflict-affected Northwest Cameroon

**DOI:** 10.1016/j.parepi.2026.e00550

**Published:** 2026-10-07

**Authors:** Calvin B. Ebai, Irene U. Ajonina-Ekoti, Joyceline B. Ndonwi, Nicoline F. Tanih, Flore N. Ngoufo, Marcelus U. Ajonina

**Affiliations:** a Department of Medical Laboratory Science, Faculty of Health Sciences, University of Bamenda, P.O. Box 39, Bambili, North West Region, Cameroon; b Department of Microbiology and Parasitology, Faculty of Science, University of Bamenda, P.O. Box 39, Bambili, North West Region, Cameroon; c Department of Medical Laboratory Sciences, Faculty of Health Sciences, PO Box 63, University of Buea, Buea, Cameroon; d Graduate School of Health and Biomedical Sciences, St Louis University Institute, Douala, Cameroon; e Division of Epidemiology and Biostatistics, Department of Global Health, Faculty of Medicine and Health Sciences, Stellenbosch University, Cape Town, South Africa; f TOZARD Research Laboratory, Bambili, North West Region, Cameroon; g McGadi Education and Research Initiative, Buea, Cameroon; h Department of Biomedical Sciences, Faculty of Health Sciences, University of Bamenda, P.O. Box 39, Bambili, North West Region, Cameroon; i School of Health Sciences, Charisma University, 2075 Overland Avenue, Billings, MT 59102, USA

**Keywords:** Intestinal protozoan infections, Risk factors, Prevalence, Asymptomatic individuals, Rural settings, Urban settings, Northwest Region, Cameroon

## Abstract

**Background:**

Intestinal protozoan infections remain an important public health problem in sub-Saharan Africa. In Cameroon, there is limited evidence comparing the epidemiology of intestinal protozoan infections in rural and urban settings. This study determined the prevalence of intestinal protozoan infections, identified associated factors, and compared selected diagnostic techniques among asymptomatic individuals in rural and urban communities of Northwest Cameroon.

**Methods:**

A school and community-based cross-sectional study was conducted between May and August 2025 in rural Bafut and urban Bamenda, Northwest Cameroon. A structured questionnaire was used to collect sociodemographic and hygiene-related data. Stool samples were analyzed using direct wet mount, formol-ether concentration, and modified Ziehl-Neelsen staining techniques. Data were analyzed using SPSS version 28. The Chi-square test or Fisher's exact test, as appropriate, and odds ratios (ORs) with 95% confidence intervals (CIs) were used to assess associations. Statistical significance was set at *p* < 0.05.

**Results:**

A total of 270 participants aged 5–63 years were enrolled. Overall, 109 (40.4%) participants were infected with at least one intestinal protozoan species. The prevalence was comparable between the rural (41.5%) and urban (39.3%) settings. *Cryptosporidium* spp. (19.6%) and *Entamoeba histolytica* (10.4%) were the most frequently detected protozoans. No statistically significant associations were observed between intestinal protozoan infection and the sociodemographic, environmental, and behavioral factors examined (*p* > 0.05). The formol-ether concentration technique yielded the highest number of positive parasite detections and identified all eight parasite species detected in the study, whereas direct wet mount and modified Ziehl–Neelsen techniques identified five and two species, respectively.

**Conclusion:**

Intestinal protozoan infections remain highly prevalent among asymptomatic individuals in conflict-affected communities of Northwest Cameroon. Improved water, sanitation, and hygiene interventions, along with the use of sensitive diagnostic techniques, may enhance control and surveillance of these infections.

## 1 Introduction

Intestinal protozoan infections (IPIs) are among the most prevalent neglected tropical diseases in sub-Saharan Africa, disproportionately affecting vulnerable populations. A systematic review and meta-analysis among some African schoolchildren revealed a moderate to high pooled prevalence of infections predominantly caused by *Entamoeba histolytica/dispar, Giardia intestinalis,* and *Blastocystis* spp. (Hajissa et al., 2022). Several studies have shown that transmission primarily occurs via the fecal-oral route through contaminated food, water, and hands due to poor sanitation and inadequate access to clean water (Damen et al., 2015; Sebaa et al., 2021; Yamssi et al., 2023). A global systematic review and meta-analysis by Badri et al. (2022) demonstrated widespread contamination of fruits and vegetables with *Giardia* spp. and *Entamoeba* spp. highlighting food safety as an important but often overlooked component of control strategies. Food handlers were identified in Nigeria as potential reservoirs for sustained community transmission (Damen et al., 2015). The greatest burden of these diseases is reported to be among children and those with low educational attainment and socioeconomic status (Hajissa et al., 2022; Smigova et al., 2022; Yamssi et al., 2023). Symptoms that have been reported among sufferers include malnutrition, growth retardation, chronic diarrhea, and impaired cognitive development, especially among children (Badri et al., 2022).

Intestinal protozoans are endemic in both rural and urban communities of Cameroon. Many communities continue to experience inadequate access to safe water and suboptimal sanitation and hygiene conditions, with reported prevalence ranging from 20% to over 30%, depending on geographic location and the diagnostic method used (Ntonifor et al., 2022). Rural and urban settings may present unique challenges related to sanitation, education levels, lifestyles, and access to healthcare. Studies in rural and urban settings have reported rapid urbanization, informal settlements, unreliable water supplies, animal ownership, and overcrowding as factors that sustain transmission in these areas (Sebaa et al., 2021; Shehab et al., 2021; Yamssi et al., 2023). Comparative data from southern Algeria revealed a significantly higher protozoan prevalence of 32.5% among symptomatic individuals than in asymptomatic populations, underscoring the clinical relevance of these infections (Sebaa et al., 2021) but also indicating that many harbor the parasites without manifesting clinically. However, comparative epidemiological data on rural and urban settings in Cameroon remain limited.

An important factor in the surveillance and control of these diseases involves accurate diagnosis. Most health facilities use wet-mount microscopy as the primary diagnostic method because of its low cost, ease of use, and affordability. However, many laboratories lack skilled technicians who can identify the presence and type of intestinal protozoa in a stool specimen. Also, when diagnosing low-intensity infections and coccidian protozoans such as *Cryptosporidium* spp. or *Cyclospora cayetanensis*, their sensitivity is widely questioned (Demeke et al., 2021). Patient management relies on an accurate diagnosis, and when this fails, it not only compromises management but also understates the actual burden of disease.

In addition, the ongoing armed conflict in the Northwest and Southwest Regions of Cameroon has disrupted healthcare delivery, education, water supply systems, and sanitation services in many communities. These disruptions may increase exposure to intestinal protozoan infections while simultaneously limiting access to diagnosis and treatment. Despite the potential public health implications of these conditions, comparative data on the prevalence, risk factors, and diagnosis of intestinal protozoan infections in rural and urban conflict-affected communities of Cameroon remain limited. These gaps underscore the need for integrated control strategies that combine improved water, sanitation, and hygiene interventions, food safety measures, health education, and the use of sensitive diagnostic techniques. This study, therefore, aimed to determine the prevalence of intestinal protozoan infections, identify factors associated with intestinal protozoan infection, and compare the performance of selected diagnostic techniques among asymptomatic individuals in rural and urban communities in conflict-affected Northwest Cameroon.

## 2 Materials and methods

### 2.1 Study design and setting

The study was carried out in two selected schools and their surrounding communities in Bamenda (urban setting) and Bafut (rural setting), from May–August 2025. The schools selected were Government Primary School Mulang-Bamenda II (GPS Mulang), and Government Primary School Nso-Bafut (GPS Nso-Bafut). The localities were selected purposively because they have been affected by the ongoing conflict in the Northwest Region of Cameroon. The schools were randomly selected from among government primary schools located in the study areas. Questionnaires were used to collect data on sociodemographic and hygiene-related factors. Stool specimens were collected and analyzed for intestinal parasites at the University of Bamenda Health Center laboratory.

Bafut is mainly rural, with crop farming and cattle rearing being the most common economic activities. Bamenda is a cosmopolitan, predominantly urban municipality. Both areas have been affected by the ongoing armed conflict in the English-speaking Regions of Cameroon. Access to potable water, healthcare, education, and other amenities has become increasingly challenging due to the physical destruction of infrastructure and disruptions in public services and healthcare delivery systems.

### 2.2 Study population and sampling

The study population comprised individuals aged 5–63 years recruited from the selected schools and surrounding communities in Bafut and Bamenda through convenience sampling. Individuals who provided consent (or, for minors, assent with parental consent) and submitted stool samples were included in the study. Individuals who declined to participate, did not provide stool specimens, or did not provide informed consent were excluded from the study.

Participants were recruited from GPS Mulang, GPS Nso-Bafut, and the surrounding communities. Recruitment was conducted during visits to the study sites, and all eligible individuals who consented to participate during the study period were enrolled.

### 2.3 Data collection

#### 2.3.1 Questionnaire administration and stool specimen collection

A pretested, structured questionnaire was administered by trained personnel to collect information on sociodemographic characteristics and hygiene-related behaviors. For children who were unable to respond independently, sociodemographic and household-level information was obtained by telephone from their parents or guardians. Single stool specimens were collected from participants using sterile containers and preserved in 10% formol saline. The specimens were transported in coolers to the University of Bamenda Health Center Laboratory for analysis.

#### 2.3.2 Laboratory procedures

In the laboratory, stool specimens were analyzed using direct wet-mount microscopy (DWM), formol-ether concentration (FEC), and modified Ziehl–Neelsen staining (MZN). For DWM, a drop of normal saline and a drop of Lugol's iodine were placed on separate glass slides. Approximately 2 mg of well-mixed stool specimen was added to each preparation, covered with a coverslip, and examined using ×10 and ×40 objectives of a light microscope (Olympus-NY) (Cheesbrough, 2005). For the FEC technique, approximately 2 mg of the stool specimen was homogenized in 0.85% physiological saline, filtered, fixed with 10% formol saline, mixed with ether, and centrifuged. The resulting sediment was examined microscopically for the detection of parasites.

For the modified Ziehl–Neelsen staining technique, approximately 30 μL of concentrated fecal sediment was smeared onto a clean glass slide, air-dried, and fixed with methanol. The smear was stained according to the modified Ziehl–Neelsen method described by Cheesbrough (Cheesbrough, 2005) and examined under oil immersion (×100) for the detection of acid-fast coccidian oocysts.

### 2.4 Data analysis

Data were entered into a Microsoft Excel spreadsheet and subsequently imported into IBM SPSS Statistics version 28 for analysis. Categorical variables were summarized using frequencies and percentages, while continuous variables were summarized using means and standard deviations. Associations between intestinal protozoan infection and sociodemographic, environmental, and behavioral factors were assessed using Pearson's chi-square test or Fisher's exact test, as appropriate. Crude odds ratios (ORs) with 95% confidence intervals (CIs) were calculated to estimate the strength of associations. Statistical significance was set at *p* < 0.05.

### 2.5 Ethical considerations

Ethical clearance for this study was obtained from the Regional Ethics Committee for Human Health Research, North West Region (No. 2025/04/38/CERSH-NW). Administrative authorizations were obtained from the Regional Delegations of Public Health and Basic Education for the Northwest Region, as well as from the University of Bamenda for the analysis of specimens in the Health Center laboratory. Participants were informed about the study's purpose, procedures, benefits, and potential risks. Written informed consent was obtained from adult participants and from parents or legal guardians of minors, while assent was obtained from minors where appropriate. Participants and their parents or legal guardians were assured that all information collected would remain confidential through the use of unique identification codes. Participation was voluntary, and participants were informed of their laboratory results. Individuals who tested positive for intestinal parasites were referred to the consulting medical officer at the District Hospital in Bafut or Bamenda, according to their place of residence.

Ethical clearance for this study was obtained from the Regional Ethical Committee for Human Health Research, North West (No. 2025/04/38/CERSH-NW). Administrative authorizations were obtained from the Regional Delegations of Public Health and Basic Education for the Northwest Region, as well as from the University of Bamenda, for the analysis of the specimens in the Health Center laboratory. Participants were informed about the study's purpose, procedure, benefits, and potential risks. Those who consented signed informed consent forms and were admitted into the study. Written informed consent was obtained from adult participants and from parents or legal guardians of minors. Assent was obtained from minors where appropriate. Participants and their parents/legal guardians were assured that all information collected would remain confidential through the use of unique identification codes. Participation was voluntary, and participants were informed of laboratory results. Individuals who tested positive for intestinal parasites were referred to the consulting medical officer at the District Hospital in Bafut or Bamenda, according to their place of residence.

## 3 Results

### 3.1 Sociodemographic characteristics of the study participants

A total of 270 participants were included in the study. The mean age of the participants was 24.5 ± 14.8 years, with ages ranging from 5 to 63 years. The majority were females (60.4%), aged 14–30 years (36.3%), had attained primary education (37.0%), and earned less than FCFA 44,000 per month (73.3%). Participants from urban settings accounted for 51.9% of the study population, while those from rural settings accounted for 48.1%. The mean household size was 6.3 ± 2.5 persons, with most participants living in households of 6–15 persons (60.7%). Pupils constituted the largest occupational group (37.0%) (Table 1).

**Table 1:** Sociodemographic Characteristics of the Study Participants.

Characteristic	Category	Overall *N* = 270, n (%)	Rural *n* = 130, n (%)	Urban *n* = 140, n (%)
Age (years)	5–13	91 (33.7)	38 (29.2)	53 (37.9)
	14–30	98 (36.3)	50 (38.5)	48 (34.3)
	31–63	81 (30.0)	42 (32.3)	39 (27.9)
Sex	Female	163 (60.4)	84 (64.6)	79 (56.4)
	Male	107 (39.6)	46 (35.4)	61 (43.6)
Education level	No formal education	30 (11.1)	16 (12.3)	14 (10.0)
	Primary	100 (37.0)	47 (36.2)	53 (37.9)
	Secondary	55 (20.4)	42 (32.3)	13 (9.3)
	Higher	85 (31.5)	25 (19.2)	60 (42.9)
Occupation	Pupil	100 (37.0)	47 (36.2)	53 (37.9)
	Student	64 (23.7)	28 (21.5)	36 (25.7)
	Professional	49 (18.1)	24 (18.5)	25 (17.9)
	Blue-collar worker	57 (21.1)	31 (23.8)	26 (18.6)
Monthly income (FCFA)	<44,000	198 (73.3)	109 (83.8)	89 (63.6)
	≥44,000	72 (26.7)	21 (16.2)	51 (36.4)
Household size	1–5	106 (39.3)	48 (36.9)	58 (41.4)
	6–15	164 (60.7)	82 (63.1)	82 (58.6)

### 3.2 Prevalence of intestinal protozoans

Out of the 270 participants who took part in this study, 109 (40.4%) were positive for at least one intestinal protozoan species detected by at least one of the diagnostic methods. There was no significant difference in prevalence across sociodemographic characteristics (*p* > 0.05). Although higher prevalences were observed among participants aged 5–13 years (41.8%), those with higher education (44.7%) and blue-collar workers (47.4%), these differences were not statistically significant (*p* > 0.05) (Table 2).

**Table 2:** Prevalence of Parasite Infection with respect to Socio-demographic Characteristics of Study Participants.

Characteristic	Category	Total examined	Positive n (%)	*p*-value
Age (years)	5–13	91	38 (41.8)	0.312
	14–30	98	34 (34.7)	
	31–63	81	37 (45.7)	
Sex	Female	163	65 (39.9)	0.841
	Male	107	44 (41.1)	
Education level	No formal education	30	11 (36.7)	0.793
	Primary	100	39 (39.0)	
	Secondary	55	21 (38.2)	
	Higher	85	38 (44.7)	
Setting	Rural	130	54 (41.5)	0.702
	Urban	140	55 (39.3)	
Occupation	Pupil	100	39 (39.0)	0.683
	Student	64	24 (37.5)	
	Professional	49	19 (38.8)	
	Blue-collar worker	57	27 (47.4)	
Monthly income (FCFA)	<44,000	198	75 (37.9)	0.162
	≥44,000	72	34 (47.2)	
Household size	1–5	106	43 (40.6)	0.963
	6–15	164	66 (40.2)	

### 3.3 Distribution of intestinal parasite species according to setting

*Cryptosporidium* spp. was the most frequently detected protozoan mono-infection (53, 19.6%), followed by *Entamoeba histolytica* (28, 10.4%) in both settings (Table 3; Fig. 1). A total of eight protozoan co-infections were identified, the most common being the combination of *Entamoeba histolytica* and *Cryptosporidium* spp. (3, 1.1%). Helminth infections occurred at low frequencies, with *Ascaris lumbricoides* detected only in the rural setting and *Taenia solium* only in the urban setting (Table 3).

**Table 3:** Distribution of Intestinal Parasite Species According to Study Setting.

Type of infection	Species	Rural n (%) (*n* = 130)	Urban n (%) (*n* = 140)	Total n (%) (*N* = 270)
Mono-infection with protozoa⁎	*Cryptosporidium* spp.	22 (16.9)	31 (22.1)	53 (19.6)
	*Entamoeba coli*	0 (0.0)	1 (0.7)	1 (0.4)
	*Entamoeba histolytica*	12 (9.2)	16 (11.4)	28 (10.4)
	*Giardia lamblia*	4 (3.1)	1 (0.7)	5 (1.9)
	*Iodoamoeba* spp.	6 (4.6)	0 (0.0)	6 (2.2)
	**Total mono-infections**	**44 (33.8)**	**49 (35.0)**	**93 (34.4)**
Co-infections between protozoa	*Cryptosporidium* spp. and *Iodoamoeba* spp.	1 (0.8)	0 (0.0)	1 (0.4)
	*Entamoeba coli* and *Cryptosporidium* spp.	2 (1.5)	0 (0.0)	2 (0.7)
	*Entamoeba coli* and *Entamoeba histolytica*	0 (0.0)	1 (0.7)	1 (0.4)
	*Entamoeba histolytica* and *Cryptosporidium* spp.	2 (1.5)	1 (0.7)	3 (1.1)
	*Giardia lamblia* and *Cryptosporidium* spp.	1 (0.8)	0 (0.0)	1 (0.4)
	**Total co-infections**	**6 (4.6)**	**2 (1.4)**	**8 (3.0)**
Other parasites	*Ascaris lumbricoides*	3 (2.3)	0 (0.0)	3 (1.1)
	*Cryptosporidium* spp. and *Ascaris lumbricoides*	1 (0.8)	0 (0.0)	1 (0.4)
	*Taenia solium*	0 (0.0)	3 (2.1)	3 (1.1)
Negative		76 (58.5)	85 (60.7)	161 (59.6)

Abbreviation: spp., multiple species within a genus.

⁎ *Mono-infection refers to infection with a single protozoan species only, without concurrent infection with another protozoan species.*

**Fig. 1:**
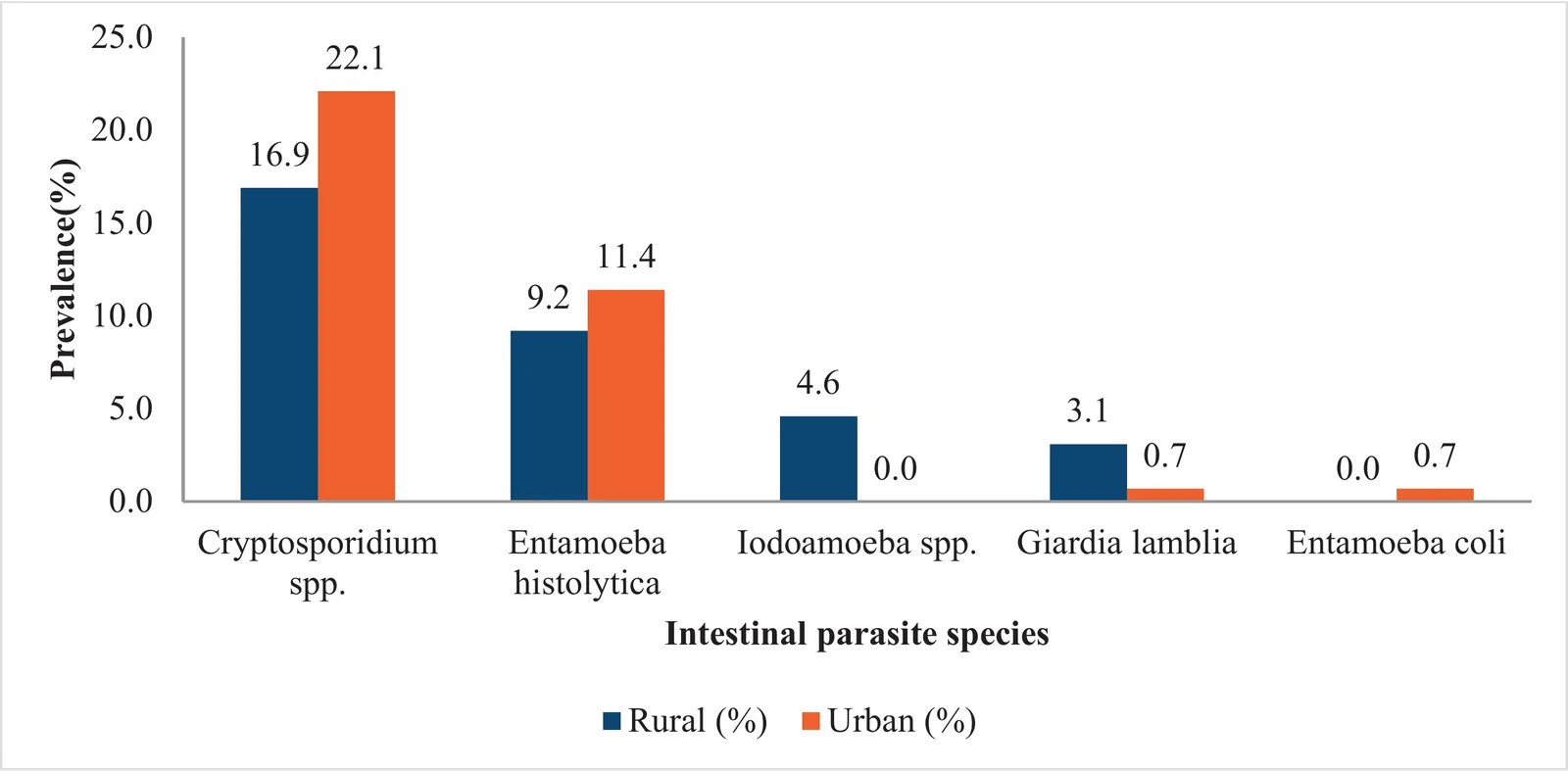
Distribution of intestinal protozoan mono-infections according to study setting.

### 3.4 Factors associated with intestinal protozoan infection

None of the factors examined was significantly associated with intestinal protozoan infection (*p* > 0.05). Infection prevalence was similar between participants from rural (41.5%) and urban (39.3%) settings. Although none of the five internally displaced participants tested positive for intestinal protozoan infection, the association between IDP status and infection was not statistically significant (Fisher's exact *p* = 0.084). No significant associations were observed between intestinal protozoan infection and household size, study setting, access to clean drinking water, drinking water treatment, presence of open defecation, washing fruits and vegetables before consumption, consumption of raw or undercooked food, ownership of pets or livestock, travel away from home, previous diagnosis of intestinal protozoan infection, or recent antiparasitic medication use (Table 4).

**Table 4:** Factors Associated with Protozoan Infection.

Factor	Category	No. tested	Positive n (%)	OR (95% CI)	p-value
Household size	1–5	106	43 (40.6)	1	
	6–15	164	66 (40.2)	0.99 (0.60–1.62)	0.958
Setting	Rural	130	54 (41.5)	1	
	Urban	140	55 (39.3)	0.91 (0.56–1.48)	0.706
IDP status	No	265	109 (41.1)	1	
	Yes	5	0 (0.0)	NE	0.084†
Access to clean drinking water	Yes	196	79 (40.3)	1	
	No	74	30 (40.5)	1.01 (0.59–1.74)	0.972
Drinking water treatment	Untreated	74	27 (36.5)	1	
	Treated	196	82 (41.8)	1.25 (0.72–2.17)	0.424
Presence of open defecation	No	106	39 (36.8)	1	
	Yes	164	70 (42.7)	1.28 (0.77–2.11)	0.335
Wash fruits/vegetables before eating	Yes	266	108 (40.6)	1	
	No	4	1 (25.0)	0.49 (0.05–4.75)	0.650†
Consume raw/undercooked food	No	52	20 (38.5)	1	
	Yes	218	89 (40.8)	1.10 (0.59–2.05)	0.755
Have pets/livestock	No	96	34 (35.4)	1	
	Yes	174	75 (43.1)	1.38 (0.83–2.31)	0.218
Traveled out of Bafut/Bamenda in the last year	No	112	43 (38.4)	1	
	Yes	158	66 (41.8)	1.15 (0.70–1.89)	0.577
Previous diagnosis of intestinal protozoan infection	No	204	86 (42.2)	1	
	Yes	66	23 (34.8)	0.73 (0.41–1.31)	0.293
Recent antiparasitic medication use	No	267	108 (40.4)	1	
	Yes	3	1 (33.3)	0.74 (0.07–8.22)	0.987†

IDP: Internally displaced persons; No.: Number; OR: Odd ratio; 1 = Reference category; †Fisher's exact test was used because of small cell counts. NE = NE = not estimable because no intestinal protozoan infection was detected among internally displaced participants.

### 3.5 Comparison of environmental and behavioral characteristics between rural and urban settings

Several environmental and behavioral characteristics differed significantly between the rural and urban settings. Rural participants were more likely to have lower monthly incomes, to report less frequent handwashing before meals and with soap after using the toilet, to consume raw or undercooked food, to own pets or livestock, and to practice open-air dumping of waste (*p* < 0.05). In contrast, untreated drinking water was more frequently reported among urban participants, and access to clean drinking water and the frequency of washing fruits and vegetables also differed significantly between the two settings (*p* < 0.05). Although all five internally displaced participants resided in the urban setting, the difference in IDP status between settings was not statistically significant (Fisher's exact *p* = 0.061). Household size, open-air defecation, washing fruits and vegetables before consumption, community clean-up measures, and previous diagnosis of intestinal protozoan infection also did not differ significantly between the rural and urban settings (*p* > 0.05) (Table 5).

**Table 5:** Distribution of environmental and behavioral characteristics according to study setting.

Factor	Category	Rural n (%) (*n* = 130)	Urban n (%) (*n* = 140)	p-value
Monthly income (FCFA)	<44,000	109 (55.1)	89 (44.9)	<0.001
	≥44,000	21 (29.2)	51 (70.8)	
Household size	1–5	48 (45.3)	58 (54.7)	0.449
	6–15	82 (50.0)	82 (50.0)	
IDP status	No	130 (49.1)	135 (50.9)	0.061†
	Yes	0 (0.0)	5 (100.0)	
Access to clean drinking water	No	27 (36.5)	47 (63.5)	0.018
	Yes	103 (52.6)	93 (47.4)	
Drinking water treatment	Treated	114(58.2)	82 (41.8)	<0.001
	Untreated	16 (21.6)	58 (78.4)	
Handwashing before meals	Always	39 (36.4)	68 (63.6)	<0.001†
	Most often	29 (35.8)	52 (64.2)	
	Rarely	56 (73.7)	20 (26.3)	
	Never	6 (100.0)	0 (0.0)	
Handwashing with soap after toilet use	Always	15 (15.6)	81 (84.4)	<0.001
	Most often	53 (57.6)	39 (42.4)	
	Rarely	54 (75.0)	18 (25.0)	
	Never	8 (80.0)	2 (20.0)	
Open-air defecation	No	49 (46.2)	57 (53.8)	0.701
	Yes	81 (49.4)	83 (50.6)	
Wash fruits and vegetables before eating	No	0 (0.0)	4 (100.0)	0.123†
	Yes	130 (48.9)	136 (51.1)	
Frequency of washing fruits and vegetables	Always	27 (29.3)	65 (70.7)	<0.001
	Sometimes	103 (57.9)	75 (42.1)	
Consume raw or undercooked food	No	13 (25.0)	39 (75.0)	<0.001
	Yes	117 (53.7)	101 (46.3)	
Have pets/livestock	No	24 (25.0)	72 (75.0)	<0.001
	Yes	106 (60.9)	68 (39.1)	
Waste disposal system	Incineration	0 (0.0)	37 (100.0)	<0.001
	Landfill	20 (54.1)	17 (45.9)	
	Open-air dumping	110 (58.5)	78 (41.5)	
	Incineration and landfill	0 (0.0)	8 (100.0)	
Community clean-up measures	Community clean-up campaigns	90 (49.5)	92 (50.5)	0.056†
	Regular waste collection	8 (40.0)	12 (60.0)	
	Community clean-up campaigns and regular waste collection	0 (0.0)	6 (100.0)	
	Regular waste collection and public awareness campaigns	0 (0.0)	2 (100.0)	
	None	32(53.3)	28(46.7)	
Diagnosed with intestinal protozoan infection previously	No	95 (46.6)	109 (53.4)	0.361
	Yes	35 (53.0)	31 (47.0)	

† Fischer's exact test.

### 3.6 Factors associated with rural residence

Participants with monthly incomes below FCFA 44,000 had approximately three times higher odds of residing in the rural setting than those earning at least FCFA 44,000 (OR = 2.97, 95% CI: 1.66–5.31; *p* < 0.001). Participants who consumed raw or undercooked food (OR = 3.48, 95% CI: 1.76–6.87; *p* < 0.001) and those who owned pets or livestock (OR = 4.68, 95% CI: 2.69–8.13; *p* < 0.001) also had significantly higher odds of rural residence. In contrast, participants who consumed untreated drinking water had substantially lower odds of rural residence than those who treated their drinking water (OR = 0.20, 95% CI: 0.11–0.37; *p* < 0.001), while participants without access to clean drinking water also had lower odds of rural residence than those with access to clean drinking water (OR = 0.52, 95% CI: 0.30–0.90; *p* = 0.018). Open-air defecation and previous diagnosis of intestinal protozoan infection were not significantly associated with rural residence (*p* > 0.05) (Table 6).

**Table 6:** Factors Associated with Rural Residence among Study Participants.

Factor	Category	OR (95% CI)	p-value
Monthly income (FCFA)	<44,000	2.97 (1.66–5.31)	<0.001
	≥44,000	1	
Access to clean drinking water	No	0.52 (0.30–0.90)	0.018
	Yes	1	
Drinking water treatment	Untreated	0.20 (0.11–0.37)	<0.001
	Treated	1	
Consume raw/undercooked food	Yes	3.48 (1.76–6.87)	<0.001
	No	1	
Have pets/livestock	Yes	4.68 (2.69–8.13)	<0.001
	No	1	
Open-air defecation	Yes	1.14 (0.70–1.85)	0.611
	No	1	
Previous diagnosis of intestinal protozoan infection	Yes	1.30 (0.74–2.26)	0.361
	No	1	
IDP status	Yes	NE†	0.061⁎
	No	1	

Abbreviations: OR, odds ratio; FCFA, Central African CFA franc; 1 = reference category; IDP, internally displaced person*.*

⁎ *NE = Not estimable because no internally displaced person was identified in the rural setting.*

### 3.7 Prevalence of intestinal protozoans with respect to diagnostic techniques

The formol ether concentration technique yielded the highest number of positive detections (120, 44.4%) and identified all eight parasite species detected in the study. The modified Ziehl–Neelsen technique detected two coccidian parasites, namely *Cryptosporidium* spp. and *Isospora* spp., with 63 positive detections (23.3%). In contrast, direct wet-mount microscopy yielded 20 positive detections (7.4%) and identified five parasite species. *Cryptosporidium* spp. was the most frequently detected parasite by both formol ether concentration and modified Ziehl–Neelsen staining, whereas *Entamoeba histolytica* was the most frequently detected parasite by direct wet mount microscopy (Table 7).

**Table 7:** Detection of Intestinal Parasite Species According to Diagnostic Technique (*N* = 270).

Parasite species	Direct wet mount n (%)	Formol ether concentration n (%)	Modified Ziehl–Neelsen stain n (%)
*Entamoeba histolytica*	13 (4.8)	33 (12.2)	0 (0.0)
*Entamoeba coli*	3 (1.1)	4 (1.5)	0 (0.0)
*Giardia lamblia*	2 (0.7)	6 (2.2)	0 (0.0)
*Iodoamoeba* spp.	1 (0.4)	7 (2.6)	0 (0.0)
*Cryptosporidium* spp.	0 (0.0)	62 (23.0)	62 (23.0)
*Isospora* spp.	0 (0.0)	1 (0.4)	1 (0.4)
*Ascaris lumbricoides*	0 (0.0)	4 (1.5)	0 (0.0)
*Taenia solium*	1 (0.4)	3 (1.1)	0 (0.0)
**Total positive detections**	**20 (7.4)**	**120 (44.4)**	**63 (23.3)**

Abbreviation: spp., multiple species within a genus.

## 4 Discussion

This study determined the prevalence of intestinal protozoan infections, identified associated factors, and compared selected diagnostic techniques in rural and urban settings affected by ongoing armed conflict. The prevalence of intestinal protozoan infections in this study was 40.4%. This is higher than the 28.1% reported by Mbuh et al., 2010 in Buea, Cameroon, the 25.9% reported by (Richardson et al., 2011a, Richardson et al., 2011b) in Western Cameroon, 31.0% reported by Cho et al., 2021 among school-aged children in Northwest Cameroon, and the 28.0% reported by Dejon-Agobe et al., 2025 in Gabon. The relatively high prevalence observed in the present study may reflect the poor environmental and sanitary conditions that characterize many conflict-affected communities. Disruptions in healthcare services, water supply, sanitation, and health education may increase exposure to intestinal protozoan infections and facilitate their transmission through the fecal–oral route.

The prevalence of intestinal protozoan infections was comparable across sociodemographic groups. This may suggest broadly similar exposure to infection across participant groups in the study area. However, non-significant differences were observed between some categories. Regarding age groups, the highest prevalence was observed in the youngest group, participants aged 5–13 years. Similar findings were reported by Mesfin et al. (2025). Children may have greater exposure to contaminated soil, water, and food because of age-related hygiene practices and frequent environmental contact. Unexpectedly, a higher prevalence was observed among participants with tertiary education. This finding should be interpreted cautiously because the association was not statistically significant and may reflect the composition of the study population rather than a true education-related risk.

A higher, but non-significant, prevalence was also observed among blue-collar workers compared with other occupational groups. The ongoing armed conflict in the English-speaking Regions of Cameroon has resulted in substantial internal displacement to areas of safety or in search of livelihood. Among the displaced are graduates from higher institutions of learning who may take up available employment, including blue-collar jobs. Some blue-collar occupations, including farming, car repair, carpentry, and waste handling, may increase environmental exposure to contaminated soil, water, or waste. Disruptions in healthcare delivery, limited access to pipe-borne water, and poor waste management in parts of the study area may further increase the transmission of fecal–oral infections among exposed populations.

Among the intestinal protozoan species detected in this study, *Cryptosporidium* spp. and *Entamoeba histolytica* were the most frequently identified mono-infections. Similar findings have been reported in Cameroon and elsewhere (Ntonifor et al., 2022; Richardson et al., 2011b; Sebaa et al., 2021). These protozoa are commonly transmitted through the fecal–oral route, especially through contaminated food, water, and hands. The slightly higher prevalence of *Cryptosporidium* spp. and *E. histolytica* in the urban setting may reflect differences in population density, food-handling practices, and environmental contamination, although these factors were not directly measured. Further studies incorporating detailed assessments of food and water exposure would help clarify these patterns. Other parasites, notably *Giardia lamblia* and *Iodoamoeba* spp., showed a higher occurrence in the rural setting, with *Iodoamoeba* spp. reported exclusively in the rural community. Higher prevalences of *Giardia lamblia* have been reported in rural areas by other researchers (Mekonnen and Ekubagewargies, 2019; Sebaa et al., 2021; Tesfaw et al., 2024). Although these parasites are cosmopolitan, their predominance in rural settings may be attributed to inadequate sanitation and poor waste disposal. In rural settings, limited access to improved sanitation facilities and greater reliance on unimproved waste disposal practices may facilitate environmental contamination and parasite transmission. Animal ownership was also more common in the rural setting, which may increase environmental exposure to zoonotic and soil-transmitted parasites where animal and human waste are poorly managed. Infection of humans and domestic animals with similar parasites has been reported in some studies(Kajero et al., 2022; Souza et al., 2023).

Findings from this study revealed co-infection with *Entamoeba histolytica* and *Cryptosporidium* spp. being the most frequently observed. Both parasites produce environmentally resistant stages that can survive under adverse conditions and may resist routine water treatment measures. These characteristics may increase their transmission in areas with poor food-handling and sanitation practices. Similar co-infections have been reported in humans elsewhere(Jupsa Mbiandou et al., 2019; Tan et al., 2022).

More co-infections were reported in rural areas than in urban areas. Hygiene and sanitation measures are often limited in rural areas due to inadequate infrastructure and poor enforcement of sanitation regulations. Human waste disposal is frequently through unimproved latrines or open-air defecation. In addition, poor management of domestic animals and animal waste may contribute to environmental contamination. These conditions may favor the spread of parasites transmitted through the fecal–oral route. The detection of *Ascaris lumbricoides* in the rural area compared with *Taenia solium* cases in the urban area is consistent with differences in environmental and food-related exposures between rural and urban settings (Bryan et al., 2020). The transmission of Ascaris primarily occurs through the fecal–oral route, whereas transmission of Taenia species is associated with the consumption of poorly cooked meat, which may be more common in urban settings where roadside meat vending is common.

Assessment of factors associated with intestinal protozoan infections revealed no statistically significant associations with the sociodemographic, environmental, or behavioral factors examined. Although internally displaced persons may experience conditions such as limited access to safe water, adequate sanitation, food, and suitable living environments that could increase their vulnerability to intestinal infections, none of the five internally displaced participants included in this study tested positive for intestinal protozoan infection. This observation should be interpreted with caution because the very small number of internally displaced participants limited the ability of the study to reliably assess an association between displacement status and infection. The absence of significant associations with the other factors examined may suggest broadly similar exposure to intestinal protozoan infections across participant groups in the study area.

Comparison of environmental and behavioral characteristics between the study sites revealed a higher prevalence of low income and several hygiene-related risks in the rural setting than in the urban setting. Urban residents may have greater access to employment, healthcare, waste disposal services, and health information, although access to these services may vary within urban communities. Factors that may compromise food hygiene, including infrequent handwashing before meals, lower rates of handwashing with soap after toilet use, consumption of raw or undercooked food, and inappropriate waste disposal practices such as open dumping of refuse, were more frequently reported among rural participants, consistent with observations from similar studies(Ihnacik et al., 2025). In contrast, untreated drinking water was more frequently reported among urban participants. Access to clean drinking water and the frequency of washing fruits and vegetables also differed significantly between rural and urban settings, whereas community clean-up measures did not. These findings indicate that environmental and behavioral characteristics varied between the two settings rather than consistently reflecting poorer hygiene conditions in either setting. Health education remains important for disease prevention, and urban residents may have greater exposure to health information through schools, healthcare facilities, radio, television, social media, and other communication channels that are more readily available in urban environments(Arshad et al., 2025; Huang et al., 2022).

Three diagnostic techniques, namely direct wet-mount microscopy, formol ether concentration, and modified Ziehl–Neelsen staining, were used in this study. Formol ether concentration yielded the highest number of positive detections and identified all parasite species detected in the study, while modified Ziehl–Neelsen staining detected only coccidian parasites. Similar findings have been reported by Endris et al., 2013, Hassan et al., 2018, and AL-Ezzy and Khadim, 2012. Although modified Ziehl–Neelsen staining and formol ether concentration are more sensitive techniques for the detection of intestinal coccidian parasites (Henriksen and Pohlenz, 1981). Direct wet mount microscopy remains widely used because it is inexpensive, simple, and accessible, particularly in resource-limited settings. Consequently, the risk of false-negative results may be substantial where more sensitive methods are unavailable.

The low sensitivity of direct wet-mount microscopy for detecting coccidian parasites may be attributed to the small amount of specimen examined compared with formol-ether concentration, which concentrates stool material, and modified Ziehl–Neelsen staining, a reference staining technique for coccidian intestinal parasites. Examining smaller stool samples reduces the likelihood of detecting parasites when parasite loads are low, especially among asymptomatic individuals. Nevertheless, parasites detected by direct wet-mount microscopy are generally easy to identify, contributing to their high specificity and continued clinical relevance in settings where more sensitive diagnostic methods are unavailable.

### 4.1 Limitations of the study

Although relatively sensitive for the detection of coccidian intestinal parasites, the modified Ziehl–Neelsen staining technique cannot differentiate oocysts to the species level (AL-Ezzy and Khadim, 2012; Nielsen and Ward, 1999). In addition, only a single stool specimen was collected from each participant; examination of multiple specimens may have increased the detection of positive cases. The small number of internally displaced persons also limits the precision of estimates for this subgroup. Finally, parasite identification was based solely on microscopy without molecular confirmation.

## 5 Conclusion

This study revealed a high prevalence of intestinal protozoan infections among asymptomatic individuals in rural and urban communities affected by the ongoing armed conflict in Northwest Cameroon. Although prevalence did not differ significantly between the two settings, several socioeconomic, environmental, and behavioral characteristics differed between rural and urban participants, indicating distinct patterns of potential exposure. *Cryptosporidium* spp. and *Entamoeba histolytica* were the most frequently detected protozoan species, while co-infections occurred more commonly in rural communities. None of the sociodemographic, environmental, or behavioral factors examined was significantly associated with intestinal protozoan infection. Among the diagnostic methods evaluated, the formol-ether concentration technique yielded the highest number of positive parasite detections and identified all parasite species detected in the study, highlighting its potential value for routine diagnosis and epidemiological investigations. Strengthening water, sanitation, and hygiene interventions, improving access to health education, and promoting appropriate diagnostic techniques may contribute to improved surveillance and control of intestinal protozoan infections in conflict-affected communities.

## CRediT authorship contribution statement

**Calvin B. Ebai:** Supervision, Methodology, Investigation, Formal analysis, Conceptualization. **Irene U. Ajonina-Ekoti:** Writing – original draft, Validation, Supervision, Methodology, Investigation, Conceptualization. **Joyceline B. Ndonwi:** Data curation, Conceptualization. **Nicoline F. Tanih:** Writing – review & editing, Writing – original draft, Methodology. **Flore N. Ngoufo:** Writing – review & editing, Validation. **Marcelus U. Ajonina:** Writing – review & editing, Writing – original draft, Validation, Software, Formal analysis.

## Consent for publication

Not applicable.

## Funding

This work received no external funding.

## Declaration of competing interest

The authors declare that they have no known competing financial interests or personal relationships that could have appeared to influence the work reported in this paper.

## Data Availability

Data supporting the write-up and conclusion of this article are provided with the articles.
